# Controlled Synthesis of ZrO_2_ Nanoparticles with Tailored Size, Morphology and Crystal Phases via Organic/Inorganic Hybrid Films

**DOI:** 10.1038/s41598-018-22088-0

**Published:** 2018-02-27

**Authors:** Ali Bumajdad, Ahmed Abdel Nazeer, Fakhreia Al Sagheer, Shamsun Nahar, Mohamed I. Zaki

**Affiliations:** 1 Chemistry Department, Faculty of Science, Kuwait University, P.O. Box: 5969, Safat, 13060 Kuwait; 2 Chemistry Department, Faculty of Science, Minia University, El-Minia, 61519 Egypt

## Abstract

In this investigation, well defined mesoporous zirconia nanoparticles (ZrO_2_ NPs) with cubic, tetragonal or monoclinic pure phase were synthesized via thermal recovery (in air) from chitosan (CS)- or polyvinyl alcohol (PVA)-ZrO_x_ hybrid films, prepared using sol–gel processing. This facile preparative method was found to lead to an almost quantitative recovery of the ZrOx content of the film in the form of ZrO_2_ NPs. Impacts of the thermal recovery temperature (450, 800 and 1100 °C) and polymer type (natural bio-waste CS or synthetic PVA) used in fabricating the organic/inorganic hybrid films on bulk and surface characteristics of the recovered NPs were probed by means of X-ray diffractometry and photoelectron spectroscopy, FT-IR and Laser Raman spectroscopy, transmission electron and atomic force microscopy, and N_2_ sorptiometry. Results obtained showed that the method applied facilates control over the size (6–30 nm) and shape (from loose cubes to agglomerates) of the recovered NPs and, hence, the bulk crystalline phase composition and the surface area (144–52 m^2^/g) and mesopore size (23–10 nm) and volume (0.31–0.11 cm^3^/g) of the resulting zirconias.

## Introduction

A key requirement of all applications of nanomaterials, especially those of metal oxide nanoparticles (NPs), is the availability of synthesis methods and techniques that facilitate tailoring of the particle size and properties. Therefore, a variety of preparation methods, including hydrothermal, combustion, gas-phase, microwave, colloidal-templating and sol-gel processing methods have, hitherto, been devised and applied for the synthesis of metal oxide NPs^1^. The most widely used of these methods is the sol-gel processing, in which the formation of metal oxide networks occurs through hydrolysis/condensation reactions of metal containing precursors in the liquid phase^2^. It has been demonstrated^3^ that the sol-gel approach can be used to prepare high purity nanomaterials in a variety of physical forms, such as porous structures, thin fibers, dense powders, thin films, and monoliths, as well as self-supporting bulk structures. Furthermore, the sol-gel method has several advantageous features not shared by the other methods, most prominently is the facile control it enables over the product composition, homogeneity and stoichiometry, and the ability to introduce dopants.

Sol-gel processing has, also, been frequently used to produce hybrid nanocomposites^4^, implementing hydrolysis/condensation of metal alkoxides, under favourable conditions, to form metal oxide network structures within polymeric matrices. Strong interfacial interactions between the matrix and dispersed metal oxide species in the hybrid have been found to be crucial in governing the distribution and size of the metal oxide NPs. Preparation of various types of polymer-metal oxide composites employing sol-gel *in situ* polymerization of metal alkoxides (encompassing silica^5^, zirconia^6^ or alumina^7^) has been described. In these cases, the generated metal oxide NPs were distributed uniformly in the matrix, which has been considered to improve their mechanical strength and thermal stability.

As a result of its excellent electrical, thermal, optical and mechanical properties, zirconium oxide (zirconia, ZrO_2_) is one of the most important ceramic materials that enjoys a wide range of diverse applications^8^. Due to its wide band gap (5–7 eV), depending on the method of preparation, zirconia is successfully implemented in the fabrication of gas sensors, metal oxide semiconductors and ceramic devices, and as fuel cell electrolyte, catalyst, catalyst support material, and thermal barrier coating^9–12^. Thermodynamically speaking, zirconia may exist in three different true polymorphs under atmospheric pressure^13,14^: (i) *monoclinic* (m-ZrO_2_) at <1150 °C, (ii) *tetragonal* (t-ZrO_2_) at 1150–2300 °C, and (iii) *cubic* (c-ZrO_2_) at >2300 °C. Each of these polmorphs may, however, exist in either of the following states^14^: metastable (often sustained by the co-existence of a true phase), stabilized (by foreign agents) and strained state (due to high temperature, or while being transformed). It is worth mentioning, that the three polymorphs have been found^15^ to assume different surface chemical properties because of their exposure of varied surface populations of hydroxyl groups, Brönsted acid and base sites, and Lewis acid/base strengths of cation/anion pair-sites. Consequently, the three polymorphs differ in their adsorption capacities, and catalytic activities and selectivities^16^. Furthermore, the different polymorphs of zirconia exhibit different mechanical/coating properties^17^. Accordingly, pure c-ZrO_2_ finds useful applications in automobile industry, and the manufacturing of oxygen sensors, fuel cells and optic fibers^18^, whereas pure t-ZrO_2_ finds applications in the manufacturing of ceramics, sensors, coatings and medical implants …etc^19^. Because of these properties and applications, which can be optimized for specific functions, different approaches have been employed^20–22^ for the selective synthesis of pure m-ZrO_2_, t-ZrO_2_, and c-ZrO_2_ phases.

Adopting theoretical and experimental approaches, Jin *et al*.^23^ showed that the thermal stability of monoclinic and tetragonal polymorphs of zirconia are particle size-dependent. They found that large particles (>30 nm) stabilize m-ZrO_2_, small particles (<14 nm) stabilize t-ZrO_2_, but a mixture of the two phases is stabilized when the ZrO_2_ particles are of intermediate size (14–30 nm). The particle size was controlled as a function of the duration of heating at 1300 °C. Jayakumar *et al*.^18^, employing reactive plasma processing, arrived at similar particle size dependency for stable m-ZrO_2_ (>20 nm) and t-ZrO_2_ (7–20 nm), but found, moreover, that c-ZrO_2_ is stabilized only when the particle size ≤6 nm. Amorphous and crystalline t-ZrO_2_ NPs (5–8 nm) were prepared using the advanced oxidation processes (AOP)/hydrothermal route^24^. Also, pure t-ZrO_2_ NPs (<5 nm) were generated using pulsed plasma in a liquid medium^25^. Cao *et al*.^26^ described the fabrication of porous t-ZrO_2_ and m-ZrO_2_ phases using a hydrothermal approach. Furthermore, a hydrothermal route was utilized to produce pure m-ZrO_2_ NPs with diameters in the range of 8–15 nm^27^.

A significant goal in this area of material science is to develop a cost-effective, simple, environmentally beneign and scalable approach for the preparation of isostructural ZrO_2_ NPs In this regard, chitosan, an aminopolysaccaride polymer (a derivative of the polysaccharide biopolymer chitin), found in nature in exoskeletons of shrimp and lobster, has been found to assume favourable properties including being biodegradable, nontoxic, inexpensive and readily acquired from marine wastes^28^. Moreover, possessed hydroxyl, amine, and carbonyl functional groups, render chitosan a material of potential affinity toward metal ion immobilization^29,30^. In a similar vein, polyvinyl alcohol (PVA), a synthetic polymer that has been used as the organic phase in the preparation of various organic/inorganic hybrid films^31^, is analogously capable of immobilizing metal ionc species due to its hydrophilicity and hydroxylation.

In the present study, we have implemented both chitosan and PVA in a simple, eco-friendly and cost-effective sol-gel processing to selectively produce zirconia NPs assuming pure or mixed crystalline phases. The method applied involves preparation of chitosan-ZrO_x_ and PVA-ZrO_x_ hybrid films, and subsequent calcination of the films to recover quatitatively the ZrOx content in the form of zirconia NPs in specific crystalline modification(s).

## Methods

### Preparation of zirconia NPs

**Figure 1:**
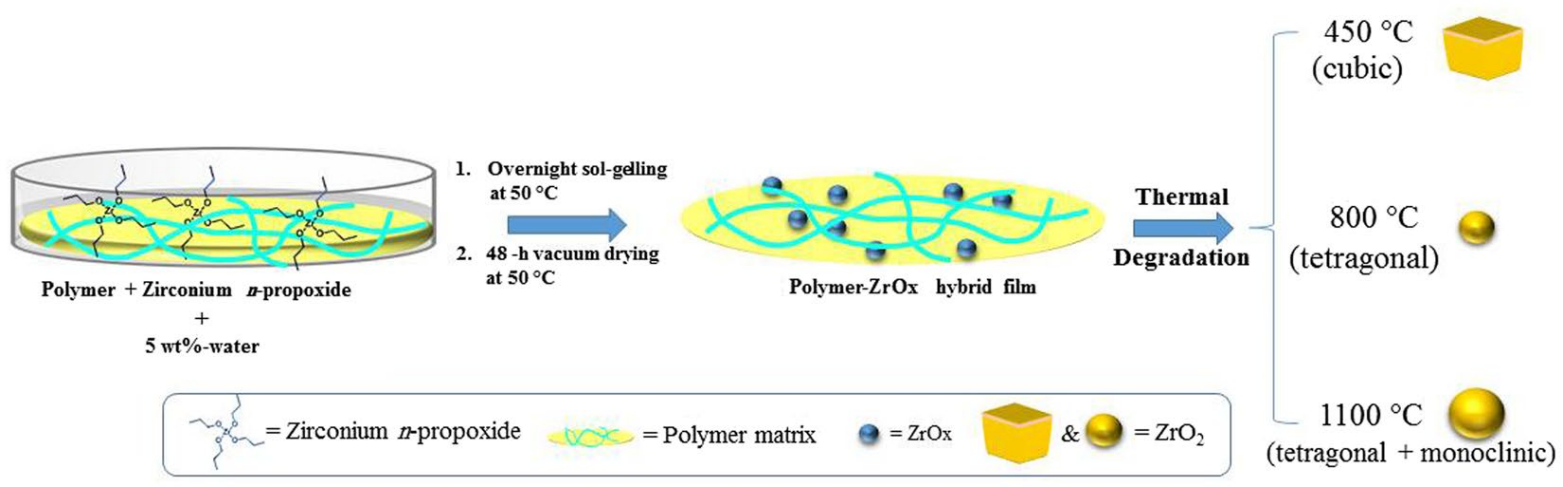
Schematic depiction of the preparation course of ZrO_2_ NPs from polymer-ZrO_x_ hybrid film.

Based on thermal analysis results (*vide* infra), ZrO_2_ NPs were obtained by calcination (in a dynamic atmosphere of air) at 450 (9 h), 800 (3 h) or 1100 °C (3 h) of hydrid films of 10 wt%-ZrO_2_ chitosan-ZrOx (CS-ZrOx) or polyvinyl alcohol-ZrOx (PVA-ZrOx), as depicted in Fig. 1. The films were prepared using Sigma-Aldrich products of medium molecular mass CS (average M_w_: 250 kDa), 99% hydrolyzed PVA (M_w_: 89,000–98,000), and 70% pure zirconium n-propoxide solution in n-propanol (M_w_: 327.57). In a typical preparation experiment, irrespective of the polymer used (CS or PVA), the polymer was dissolved in a 2 wt%-acetic acid solution in deionized water, in order to produce a 2 wt%-polymer solution. The resulting solution was stirred for 48 h at room temperature (RT) to warrant homogeneity. Then, required amount of the solution was placed in a 50-mL bottle, and appropriate amount of Zirconium n-propoxide was added to it, followed by 1 h stirring at RT. In this work, the water/Zr-propoxide molar ratio was kept as 1:4. Based of that, the required amount of an equimolar mixture of n-propanol and water was added to the solution (each film need 0.153 g). This was followed by overnight stirring at RT to promote the sol-gel processing^32^. The resulting mixture was, then, cast in Teflon Petri dish and, because of solvent elution, thin film was obtained. Both types of film (CS-ZrOx and PVA-ZrOx) with the same amount of zirconia (10 wt %) in the polymer matrix were dried at 50 °C for 18 h and, then, left under vacuum for 48 h at the same temperature, for complete removal of solvent. Eventually, flexible hybrid films yielded were calcined at the temperatures, and for the time durations, above specified. The produced zirconia nanopowders were sieved through a 63 µm mesh size, and kept dry till further use.

### Bulk characterization of zirconia NPs

Calcination temperatures applied to obtain ZrO_2_ NPs were specified by means of thermogravimetric (TG) analysis of the hybride films. TG curves were obtained upon heating (10 °C/min) 10 mg portion of test sample up to 1000 °C in a dynamic atmosphere (50 cm3/min) of synthetic air, using a model TA-50 Shimadzu thermal system (Japan) with in line work station. Chemical and phase compositions, as well as average crystallite sizes, were determined for the calcination products emplying Fourier-transorm infrared (FT-IR) spectroscopy, X-ray powder diffractometry (XRD) and Laser Raman (LRa) spectroscopy. IR spectra were taken (at 4000–400 cm^−1^, and the resolution of 4 cm^−1^) from (≤1 wt%) KBr-supported thin discs of test samples, using a model 2000 Perkin-Elmer FT-IR spectrometer (USA). X-ray powder diffractograms were recorded (at 2θ = 10–80° and RT) using a model D500 Siemens diffractometer (Germany) equipped with Ni-filtered CuKα radiation (λ = 0.15406 nm). The diffractometer was operated with 1° diverging and receiving slits at 50 kV and 40 mA, and continuous scans with a step size of 0.014° and a step time of 0.2 s. An online automatic search system was used in accordance with JCPDS standards^33^. Crystallite sizing was performed adopting the line-broadening technique and Sherrer’s formula^34^. Raman spectra of the samples were measured using Renishaw inVia Raman Microscope with He-Ne laser (632.8 nm) as the excitation source.

### Surface characterization of zirconia NPs

Surface texture (specific area and porosity), chemical composition, morphology, and microstructure of ZrO_2_ NPs were assessed by means of N_2_ sorptiometry, X-ray photoelectron spectroscopy (XPS), and transmission electron (TEM/HRTEM) and atomic force (AFM) microscopy. N_2_ adsorption-desorption isotherms were determined on degassed (at 100 °C and 10^−3^ Torr) test samples for 12 h befor the analysis at liquid nitrogen temperature (−195 °C), using a model ASAP 2010 automatic Micromeritics sorptiometer (USA), equipped with a degassing platform and an on-line data acquisition and handling system powered with BET (Brunauer–Emmett–Teller^35^) and BJH (Barrett-Joyner –Halenda^36^) based analytical software for adsorption data analysis. XPS spectra were recorded on a model VG Scientific 200 spectrometer (UK) using MgKα radiation (1253 eV) and operated at 23 kV and 13 mA. All electron binding energies were determined with respect to the C(1 s) line (284.6 eV) originating from adventitious carbon, and standard deviations of the peak positions were estimated to be ± 0.2 eV. TEM micrographs were obtained on a JEOL JEM 1230 microscope (Japan) operated at 120 KV, HRTEM micrographs on a JSM-3010 JEOL ultra high-resolution electron microscope (Japan) operated at 300 kV and 119 μA, and AFM micrographs on a model VEECO-Nano-scope IV multiple AFM/SPM microscopy (USA).

## Results and Discussion

### Thermal recovery of zirconia NPs

TG curves obtained for CS-ZrOx and PVA-ZrOx hybrid films are compared to those given rise by the pure CS and PVA polymers in supplementary Fig. S1 (available on line). It is obvious from the figure that TG curves of the pure CS and PVA indicate that their stepwise thermal decomposition results in 100% mass loss at ≥500 °C. In contrast, the stepwise thermal decomposition monitored for the two films leads to mass invariant solid residues amounting to ca. 10%, which is almost identical to the zirconia content (10%) of CS-ZrOx or PVA-ZrOx. Based on literature data, the three mass loss steps (I-III) monitored for CS-ZrOx are due to decomposition of low molecular weight species maximized at 273 °C^32^, dehydration/depolmerization of CS (maximized at 527 °C)^37^, and decomposition of minority residual species (maximized at 627 °C)^38^. On the other hand, the two mass loss steps (I and II) monitored for PVA-ZrOx are assignable to PVA dehydration into a polyacetylene-like structure (maximized at 320 °C), and decomposition of byproduct thus formed from PVA main chain (maximized at 450 °C)^39^. Accordingly, isothermal heating at ≥450 °C in air (for up to 9 h) of either of the two hybrid films should warrant quantitative thermal recovery of their ZrOx contents.

In order to reveal nature of the resulting solid residues of thermal decomposition of CS-ZrOx and PVA-ZrOx, their calcination products at 450 (for 9 h), 800 (3 h) and 1100 °C (3 h) were examined by IR spectroscopy. IR spectra thus obtained were found largely similar; therefore, the IR spectrum obtained for the 450 °C calcination product of CS-ZrOx is shown for sake of representation in supplementary Fig. S2. The spectrum monitors a medium, broad absorption at 3430 cm^−1^, two weak absorptions at 1633 and 1349 cm^−1^, and a very strong, broad absorption maximized at 493 cm^−1^. The latter absorption is indicative of νZr-O bond vibrations in crystal lattices of bulk ZrO_2_^40^. Whereas, the absorption at 3430 cm^−1^ is due to νOH vibrations of hydrogen-bonded surface-OH groups^41^, but that at 1633 cm^−1^ is diagnostic to δOH vibrations of freely bending H_2_O molecules^41^. The minute absorption at 1349 cm^−1^ is most likely assignable to νOCO vibrations of impurity carbonate surface species^42^. Accordingly, the calcination products of CS-ZrOx and PVA-ZrOx films at ≥450 °C are predominantly made up of bulk zirconia, whose particles expose surface OH and carbonate species and excess water molecules.

### Bulk characteristics of zirconia NPs

**Figure 2:**
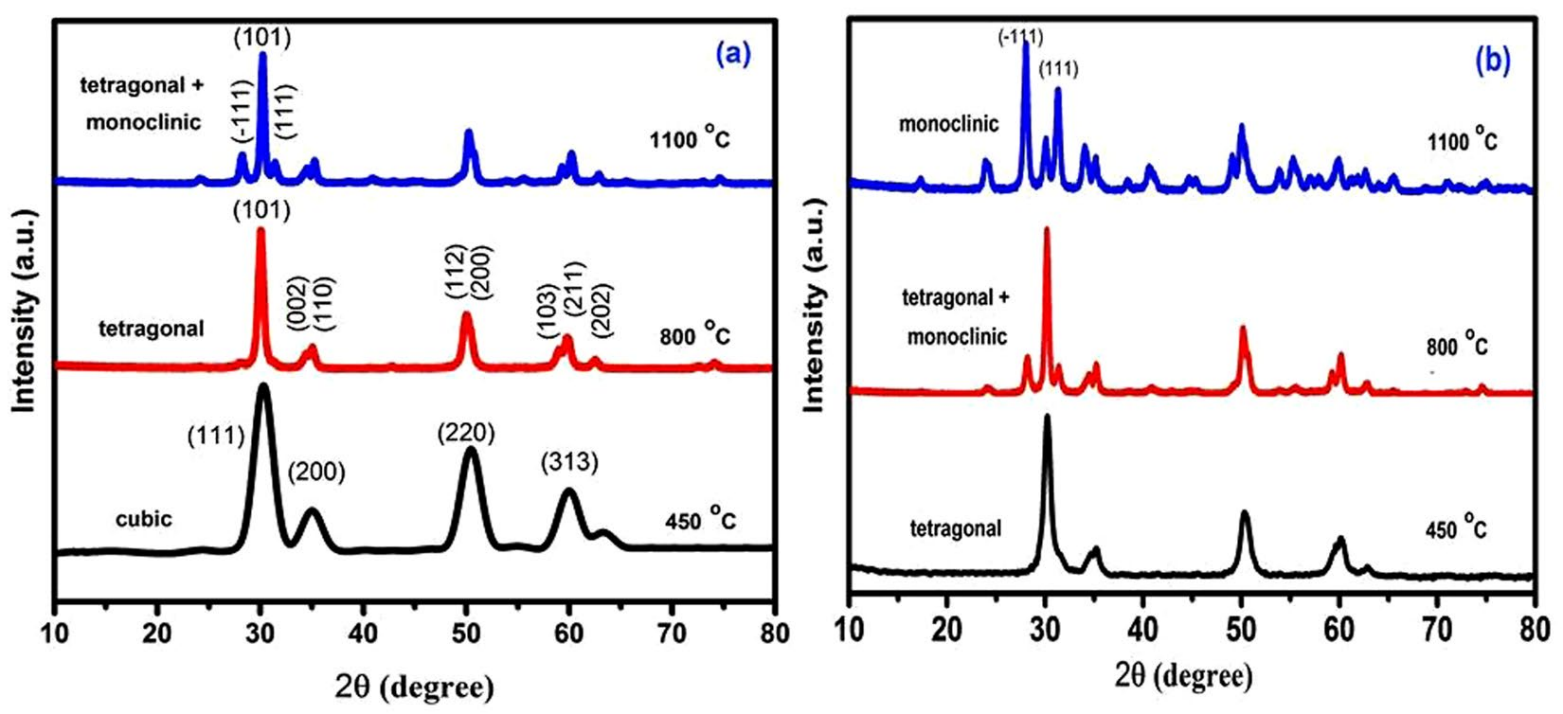
X-ray powder diffractograms obtained for calcination products of CS-ZrO_x_ (**a**) and PVA-ZrO_x_ (**b**) hybrid films at the temperatures indicated.

Calcination products of CS-ZrOx and PVA-ZrOx at 450, 800 and 1100 °C were subjected to X-ray diffractmetry in order to determine crystallite phase composition and average size, and results obtained are shown in Fig. 2(a and b) and Table 1. The diffractogram obtained for the 450 °C calcination product of CS-ZrOx (Fig. 2a) displays broad diffraction peaks at 2θ = 30.3, 35.14, 50.48 and 60.2°, which match closely those filed for c-ZrO_2_ in JCPDS 27–0997^33,43^. The diffractogram obtained for the calcination product at 800 °C (Fig. 2a) exhibits a diffraction pattern that may look generally similar to that displayed in the previous one, but it is distinct by monitoring detectably sharper peaks and resolves fine structures near 2θ = 35 and 60°. According to JCPDS 80–0965^33^, these modifications are diagnostic to formation of larger crystallites of t-ZrO_2_ (Table 1). On the other hand, the diffractogram obtained for the calcination product of CS-ZrOx at the higher temperature of 1100 °C is shown (Fig. 2a) to maintain similar, though narrower, peaks due to larger crystallites of t-ZrO_2_, and to monitor, furthermore, new peaks at 2θ = 28.2 and 31.4° that are quite indicative for formation of m-ZrO_2_ crystallites (as per JCPDS 37–1484)^33^. The derived average crystallite sizes (Table 1) allocate the smallest value (5.0 nm) to c-ZrO_2_, and the relatively largest value (20.3 nm) to m-ZrO_2_, whereas t-ZrO_2_ is shown to exist in crystallites of intermediate sizes (8.1–16.2 nm). These results are consitent with the size dependency limits of stability of each of three phases^44^. Earlier reports^13,14,18,23^ focusing on the size dependence of structural stability in ZrO_2_ particles, make clear that for nano-sized particles (where the surface/size ratio is high) the surface free energy has the upper hand in stabilizing the otherwise unstable c-ZrO_2_ at low temperature, particularly in the absence of foreign ion dopants as in the present case.

**Table 1:** Phase composition, average particle size (a), BET specific surface area (S_BET_), and BJH pore volume (v_p_) and size (d_p_) derived for calcination products of data of ZrO_2_ NP’s formed from CS-ZrO_x_ and PVA-ZrO_x_ hybrid films at 450, 800, and 1100 °C.

Film	Calcination temp./°C	Crystallite phase	a_XRD_^a^ /±0.2 nm	a_TEM_^b^ /±2 nm	S_BET_ / ± 3 m²/g	ν_p_ ± 0.02 cm³/g	d_p_ ± 2 nm
CS-ZrOx	450	c	5.0	6	144	0.31	10
	800	t	8.1	12	83	0.24	13
	1100	t + m	16.2 + 20.3	22	56	0.19	18
PVA-ZrOx	450	t	7.5	9	92	0.26	14
	800	t + m	15.8	18	65	0.18	16
	1100	m	29.2	30	52	0.11	23

^a^XRD-determined average crystallite size.

^b^TEM-determined average particle size.

Figure 2b stacks X-ray diffractograms obtained for calcination products of PVA-ZrO_x_. The diffraction pattern recorded for calcination product at 450 °C is similar to that of the CS-ZrOx calcination product at 800 °C (Fig. 2a) in assigning the formation of t-ZrO_2_^45^. Whereas that obtained for the calcination product of PVA-ZrOx at 800 °C (Fig. 2b) is similar to the pattern given rise by the calcination product of CS-ZrOx at the higher temperature of 1100 °C (Fig. 2a) in accounting for formation of mixed t-/m-ZrO_2_. Hence calcination of PVA-ZrOx at the the higher temperature of 1100 °C is found (Fig. 2b) to advance the transformation of t-ZrO_2_ into m-ZrO_2_, which is the sole detectable crystallite phase for the zirconia nanoparticles thus recovered. It is obvious from Table 1, that the average crystallite sizes determined for PVA-ZrOx calcination products at 450 (7.5 nm), 800 (15.8 nm) and 1100 °C (29.2 nm) may account for the failture in sustaining c-ZrO_2_ phase at 450 °C (size >5 nm), as well as for the instability of t-ZrO_2_ in favour of m-ZrO_2_ at 1100 °C (crystallite size approaching 30 nm)^44^. These findings suggest convincingly that the average crystallite size and, hence, the relative stability (or metastability) of the three crystallite phases of the recovered zirconia NPs are dependent not only on the thermal recovery conditions applied, but also on the polymer used in the hybrid film.

**Figure 3:**
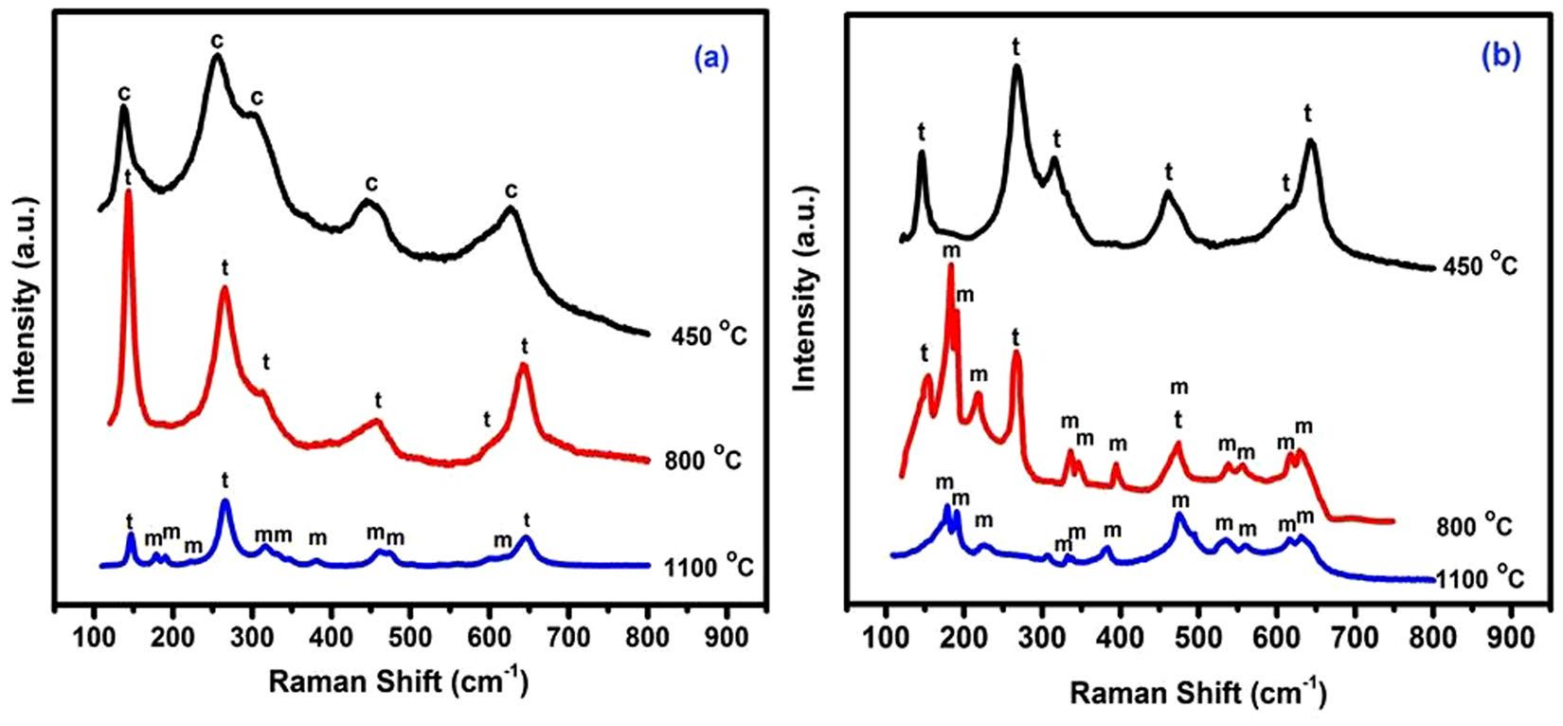
Ra spectra obtained for calcination products of CS-ZrO_x_ (**a**) and PVA-ZrO_x_ (**b**) hybrid films at the indicated temperatures.

Raman spectroscopy was used to examine further calcination products of the two hybrid films, because its detectability is, unlike X-ray diffractometry, almost particle size independent. However in line with the XRD results (Fig. 2a), the broad, ill-defined peaks displayed (at 246, 303, 444 and 617 cm^−1^) in the Ra spectrum of the zirconia NPs recovered from CS-ZrOx at 450 °C (Fig. 3a) are, according to Gazzoli *et al*.^46^, due solely to c-ZrO_2_. It is worth emphasizing, that peaks attributable to t-ZrO_2_, which predominate the Ra spectrum of the NPs recovered at 800 °C (Fig. 3a), are not resorvable amongst the c-ZrO_2_ peaks. In fact, Ra peaks diagnosing t-ZrO_2_ are discerned from those of c-ZrO_2_ not only by their detectably different frequencies (like 265, 316, 459, 600 and 643 cm^−1^, Fig. 3b)^45^, but also by their better definition. Ra spectrum of zirconia NPs recovered from CS-ZrOx at the higher temperature of 1100 cm^−1^ reveals that t-ZrO_2_ remains the major phase together with a co-existing minor phase of m-ZrO_2_ (Fig. 3a). This may be ascribed to the crystallite size (Table 1) being still in the size range (<30 nm) that can stabilize t-ZrO_2_ versus transformation into the monoclinic phase^18,23^. Inspection of Ra spectra given rise by zirconia NPs recovered from the PVA-ZrO_x_ hybrid films (Fig. 3b) reveals that characteristic peaks of t-ZrO_2_ predominate the spectrum of the nanopartices recovered at 450 °C, in an obvious contrast to the spectrum of those recovered at the same temperature from CS-ZrOx where Ra peaks monitored (Fig. 3a) diagnose sole formation of c-ZrO NPs. The thermal recovery at 800 °C is found to produce NPs giving rise to Ra peaks assignable to coexisting t-/m-ZrO_2_ (Fig. 3b). Upon further increase of the calcination temperature up to 1100 °C, NPs recovered exhibit characteristic Ra peaks (at 179, 189, 222, 333, 337, 381, 477, 501, 537, 558, 615 and 631 cm^−1^, Fig. 3b) that are due solely to m-ZrO_2_^11^. Hence, Ra spectroscopy results are in line with the XRD results, a fact that means that the recovered zirconia NPs did not contain phases other than those detected by XRD.

### Surface characteristics of zirconia NPs

**Figure 4:**
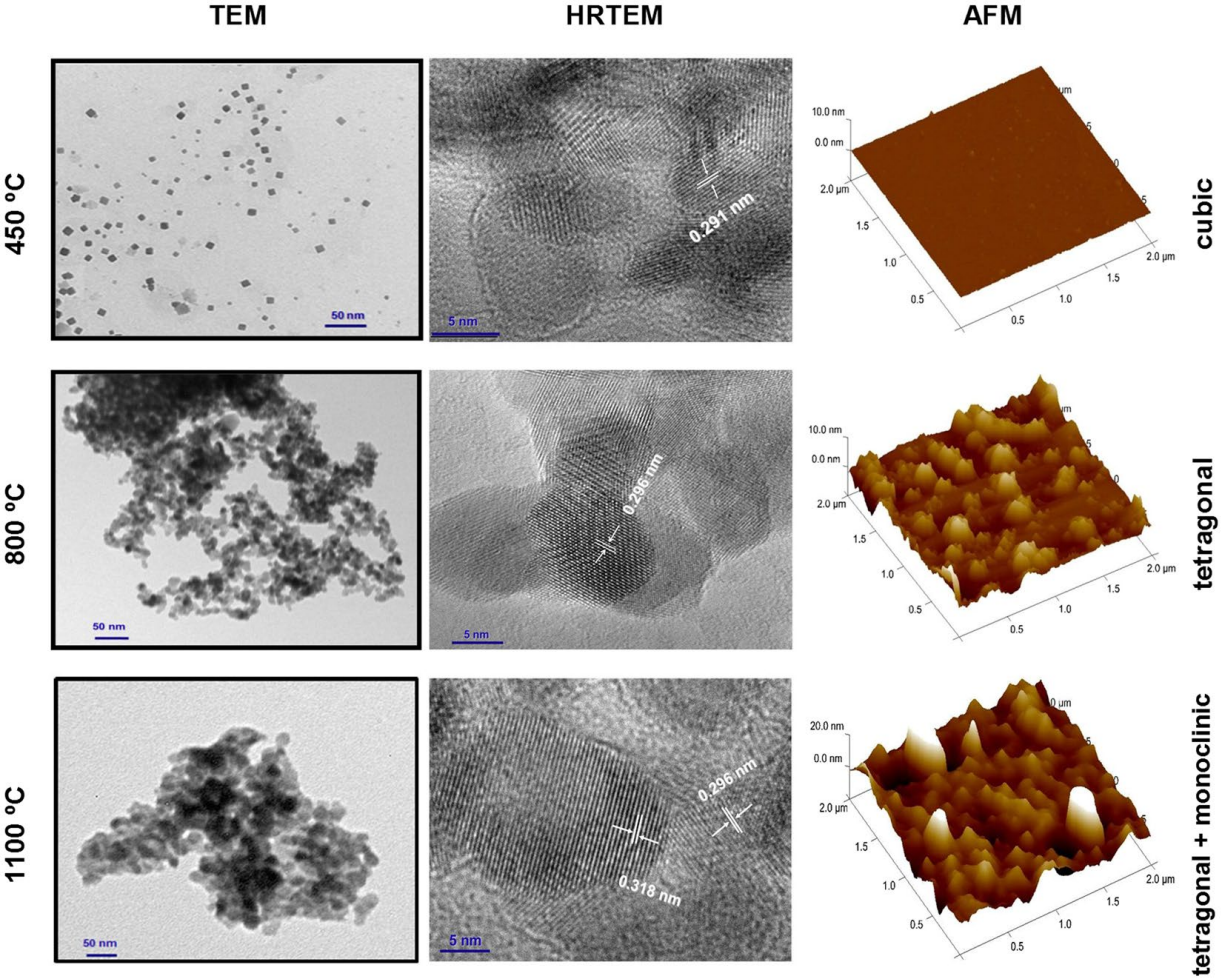
TEM, HRTEM and AFM images obtained for ZrO_2_ NPs recovered from CS-ZrO_x_ hybrid film at the calcination temperatures indicated.

TEM, HRTEM and AFM microprographs obtained for ZrO_2_ NPs recovered from CS-ZrOx and PVA-ZrOx films at 450–1100 °C are displayed respectively in Fig. 4 and the supplementary Fig. S3. Figure 4 visualizes isolated squared sections of cubic lattices of c-ZrO_2_ recoverd from CS-ZrOx at 450 °C (TEM), and resolves, consistently, crystal fringes spaced with 0.291 nm (HRTEM) that reveals the surface exposure of the closely packed (111) crystal facet of c-ZrO_2_^33,43^. The nearly smooth surface topography visualized (AFM) may be attributed to the presence of uniformerly cubic-shaped nanopartcles of very small sizes as determined by XRD (5.0 nm) and TEM (6.0 nm) (Table 1). The fact that the TEM-determined particle size is very close to the XRD-determined one may reveal that the recovered c-ZrO_2_ NPs are single crystallite particles. This may explain the high quality TEM imaging of isolated squared sections of cubic particles. The micrographs obtained for t-ZrO_2_ NPS recovered at 800 °C from CS-ZrOx (Fig. 4) display particle coalescences having surfaces exposing crystal fringes spaced with 0.296 nm, which diagnose the exposure of the loosely packed (101) facet of the tetragonal lattice^33,43^. The surface topography communicated (AFM) is shown to be non-smooth, probably due to the observed non-uniform coalescence of relatively larger NPs (12 nm, Table 1) than those of c-ZrO_2_ recovered at 450 °C (6 nm, Table 1). The larger TEM-determined particle size (12 nm) than the XRD-determined one (8.1 nm) may be considered consistent with the observed particle coalescence (TEM) rendering the particles single crystallite no longer. For the t-/m-ZrO_2_ NPs recovered at 1100 °C from CS-ZrOx, the TEM micrograph (Fig. 4) declares formation of particle agglomerates exposing crystal fringes (HRTEM) spaced with not only 0.296 nm, which corresponds to (101) facets of t-ZrO_2_, but also with 0.318 nm thus accounting for the co-exposure of (-111) facet of m-ZrO_2_^33,43,47^. Moreover, a rougher surface topography consitituting two different morphologies (small and large round-headed particles) is displayed in the AFM micrograph (Fig. 4).

Within the above context, particle morphological features and surface microstructure and topography displayed for zirconia NPs recovered from PVA-ZrOx in the TEM, HRTEM and AFM micrographs shown in the supplementary Fig. S3 on line are compatible with the formation of t-ZrO_2_ at 450 °C, t-/m-ZrO_2_ at 800 °C and m-ZrO_2_ at 1100 °C. Moreover, the TEM-determined particle sizes (9, 18 and 30 nm, respectively; Table 1) are consolidative to the stabilities of the formed zirconia phases^18,23^.

**Figure 5:**
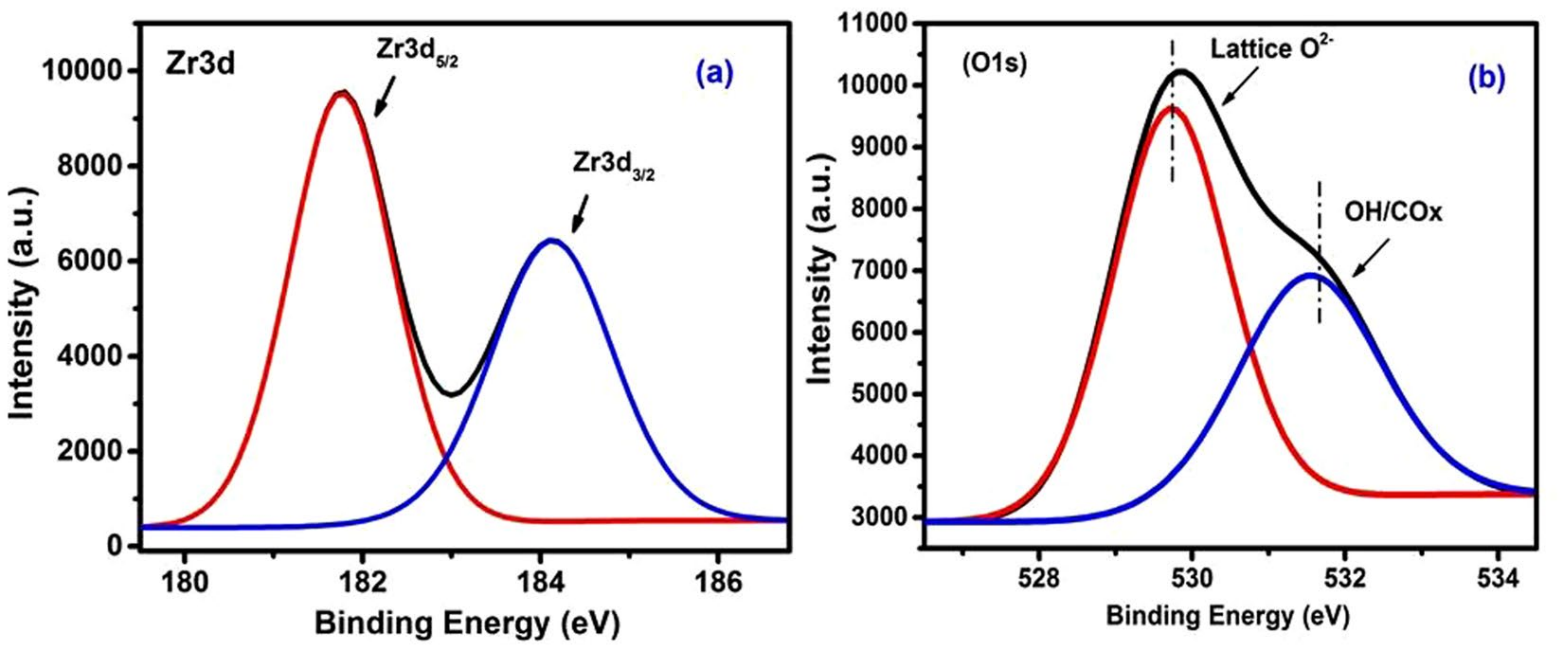
Deconvoluted XPS Zr3d (**a**) and O1s (**b**) spectra obtained for ZrO_2_ NPs recovered from CS-ZrO_x_ hybrid film at 450 °C.

Surface chemical composition of recovered ZrO_2_ NPs was probed by X-ray photoelectron spectroscopy. Full XPS spectra obtained for the entire set of test samples were similar in probing the sole presence at the surface of Zr and O atoms, as well as minority C atoms associated most likely with adventitious CH_x_/CO_x_ species. Zr3d and O1s photoelectron emission spectra were quite similar in resolving two binding energy different emission peaks in each set of spectra. BE values thereform determined are cited in supplementary Table S1. For representation, the spectra obtained for c-ZrO_2_ NPs recovered at 450 °C from CS-ZrOx film are exhibited in Fig. 5(a,b). Binding energy values (182.0 and 184.4 eV) and separation (2.4 eV) determined in the Zr3d spectrum (Fig. 5a) are due, respectively, to Zr3d_5/2_ and Zr3d_3/2_ emissions of Zr^4+^ sites^48^. Whereas, binding energy values (530.0 and 531.5 eV) determined in the O1s spectrum (Fig. 5b) are assignable, respectively, to lattice oxide (O^2−^) sites and the oxygen of OH/H_2_O/CO_x_ surface species^48^. Table 1 shows that irrespective of the hybrid film used and the calcination temperature applied, binding energies determined for Zr3d (182.0–181.7 eV for Zr3d_5/2_ and 184.4–184.3 eV for Zr3d_3/2_) and O1s (530.0–529.7 eV for O^2−^ and 531.5–531.3 eV for OH/H_2_O/CO_x_) occur in narrow ranges assignable to surfaces exposed on ZrO_2_ lattice with minority oxygen-containing surface groups (OH/CO_x_)^41,48,49^. Accordingly, surfaces of the entire set of ZrO_2_ NPs recovered are composed dominantly of Zr^4+^ and O^2−^ sites, as well as minority surface groups of OH and CO_x_ species, and water molecules. Such minority species were also identified in corresponding IR spectra (Fig. S2). These results prove that the recovered ZrO_2_ NPs expose rather chemically clean surfaces.

Surface texture (specific area and porosity) of recovered ZrO_2_ NPs was assessed by BET and BJH analyses of N_2_ adsorption-desorption isotherms thereon determined. Isotherms determined on ZrO_2_ NPs recovered at 450–1100 °C from CS-ZrOx and PVA-ZrOx films are shown respectively in supplementary Fig. S4(a and b). Inset figures display corresponding BJH pore volume distribution (pvd) curves. Data analysis results derived from the isotherms are set out in Table 1. All of the isotherms determined are similar of being of type-IV and in encompassing type-H3 hysteresis loops^50^. Hence, surfaces of the recovered nanoparticle zirconias, irrespective of the source hybrid film and the calcination temperature applied, contain slit-shaped mesopores^50^. Table 1 shows that pore size increases (from 10.4 up to 18.3 nm for zirconias recovered from CS-ZrOx, and from 13.6 up to 22.5 nm for those recovered from PVA-ZrOx), but the pore volume decreases (from 0.31 down to 0.19 cm^3^/g and from 0.26 down to 0.11 cm^3^/g, respectively), upon increasing the calcination temperature (from 450 up to 1100 °C), irrespective of the source film. Correspondingly, the specific surface is shown (Table 1) to decrease from 144 down to 56 m^2^/g and from 92 down to 52 m^2^/g. Recalling the TEM-observed particle size increase (Figs 4 and S3) as a result of heat-induced particle coalescence and subsequent agglomeration, upon increasing the calcination temperature, one may plausibly suggest that the mesopores are dwelled in inter-particle voids whose size would change with the particle size^50^ and packing.

These results may reveal that the surface textural characteristics of the present nanoparticle zirconias are relevant to the particle morphological features and consequences of their recovery thermal conditions. As regards influence of the source hybrid film, results set out in Table 1 may help implying that films made using the natural chitosan polymer yield ZrO NPs of relatively higher surface area than those yielded using the synthetic PVA polymer. However, the larger rate of loss on surface area exhibited by the former set of ZrO_2_ NPS, upon increasing the calcination temperature, may help inferring that PVA-ZrOx filmes yield less sinterable NPs than the nanoparticles recovered from the CS-ZrOx films. In all cases, however, the present ZrO_2_ NPs assume more accessible surfaces than those prepared by other methods^51,52^.

## Conclusion

The present investigation paves the way to the development of a low-temperature, cost-effective and environmentally beneign sol-gel method, whereby zirconium n-propoxide is quantitatively retained by natural chitosan (CS) or synthetic polyvinal alcohol (PVA) polymer in the form of organic-inorganic hybrid films. A subsequent thermal degradation of either of the two film types in air converts quantitatively its ZrOx content into nanoparticle zirconia (ZrO_2_) of specific crystalline phase composition, depending on the thermal recovery condition applied. Accordingly, pure cubic-ZrO_2_ NPs (6 nm, S_BET_ = 144 m^2^/g) are recoverd after calcination of CS-ZrOx film at 450 °C, but pure monoclinic-ZrO_2_ NPs (30 nm, S_BET_ = 52 m^2^/g) is yielded after calcination of PVA-ZrOx film at 1100 °C. Whereas tetragonal-ZrO_2_ NPs sized 9-nm (S_BET_ = 92 m^2^/g) are produced after calcination of PVA-ZrOx at 450 °C, or sized 12-nm (S_BET_ = 83 m^2^/g) after calcination of CS-ZrOx at 800 °C. Surfaces of the recovered ZrO_2_ NPs are solely chemically composed of Zr^4+^ and O^2−^ sites, with minority OH/CO_x_ species, irrespective of the source film used and calcination temperature applied. Stability of the surface textural properties (144–52 m^2^/g and 23–10 nm sized mesoporosity) to heating at up to 1100 °C may render the method devised a potential preparation course for catalytic-grade ZrO_2_ NPs of tailore bulk crystalline phase composition and surface texture.

## Electronic supplementary material

Supplementary Information
