# Supplementary material for: Controlled Synthesis of ZrO2 Nanoparticles with Tailored Size, Morphology and Crystal Phases via Organic/Inorganic Hybrid Films
Source: Sci Rep. 2018 Feb 27;8:3695. doi: 10.1038/s41598-018-22088-0 (PMC5829248; doi:10.1038/s41598-018-22088-0)
Supplement: Supplementary file 1 — Supplementary Information [file 41598_2018_22088_MOESM1_ESM.pdf]

Supplementary Information File

Controlled Synthesis of ZrO_2 Nanoparticles with Tailored Size, Morphology and Crystal phases via Organic/Inorganic Hybrid Films

Ali Bumajdad[a]*, Ahmed Abdel Nazeer[a], Fakhreia Al Sagheer[a], Shamsun Nahar[a], Mohamed I. Zaki[b]

[a]*Chemistry Department, Faculty of Science, Kuwait University, P.O. Box: 5969, Safat 13060, Kuwait.*

[b]*Chemistry Department, Faculty of Science, Minia University, El-Minia 61519, Egypt.*

* **Corresponding Author:**

Ali Bumajdad

Professor of Physical chemistry

Chemistry Department,

Faculty of Science,

Kuwait University

P.O. Box 5969,

Safat 13060,

Kuwait

E-mail: a.bumajdad@ku.edu.kw

Results and discussion

Thermal genesis of zirconia NPs

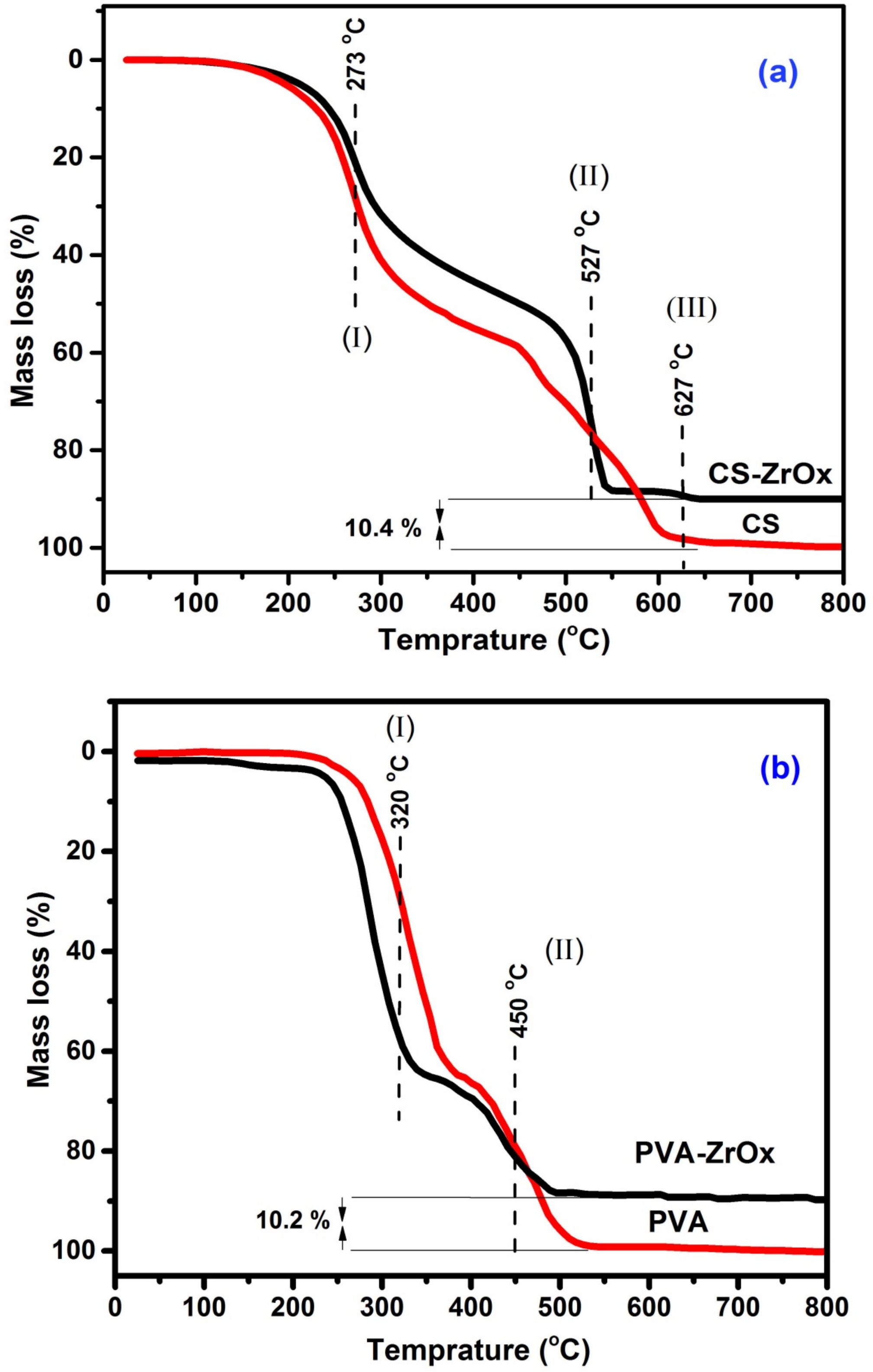

Figure S1 TG curves obtained for (a) CS-ZrOx and pure CS, and (b) PVA-ZrOx and pure PVA, upon heating test samples at 10 °C/min in a dynamic atmosphere (50 cm^3/min) of synthetic air.

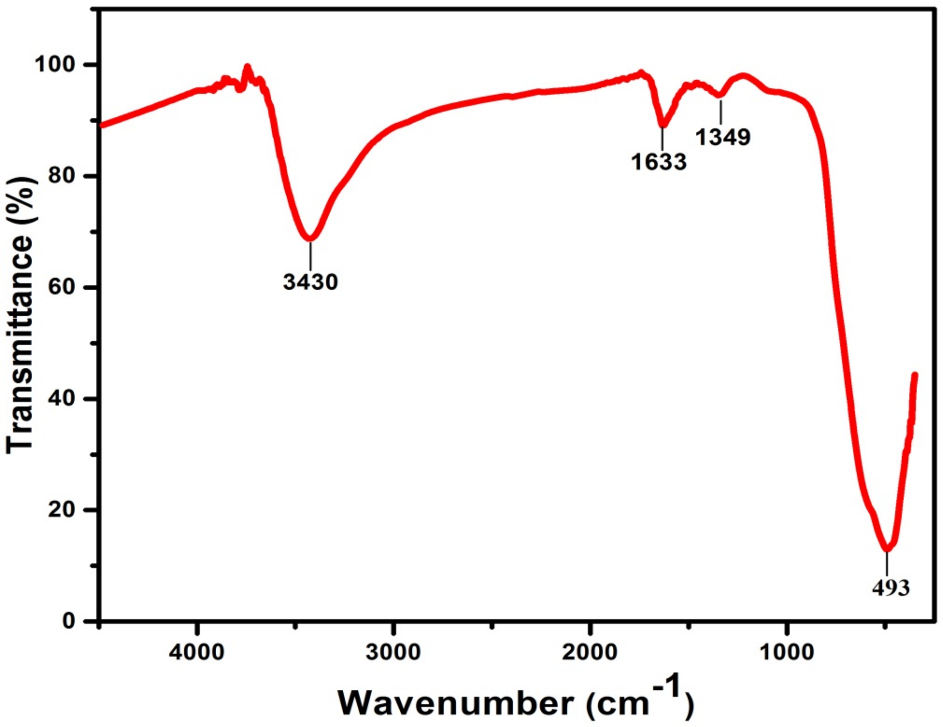

Figure S2 FT-IR spectrum obtained for the calcination product of CS-ZrO_x hybrid film at 450° C for 9 h.

Surface characteristics of zirconia NPs

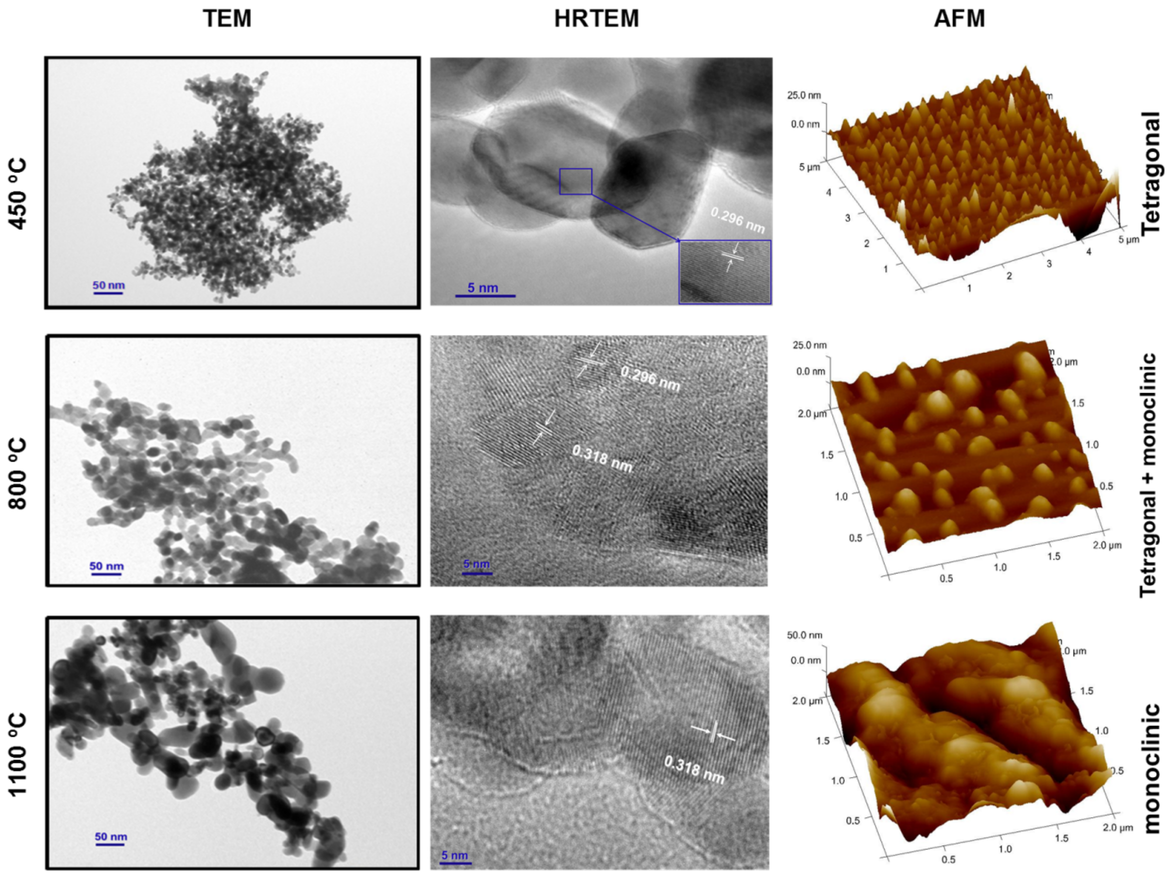

Figure S3. TEM, HRTEM and AFM images obtained for ZrO_2 NPs recovered from PVA-ZrO_x hybrid film at the calcination temperatures indicated.

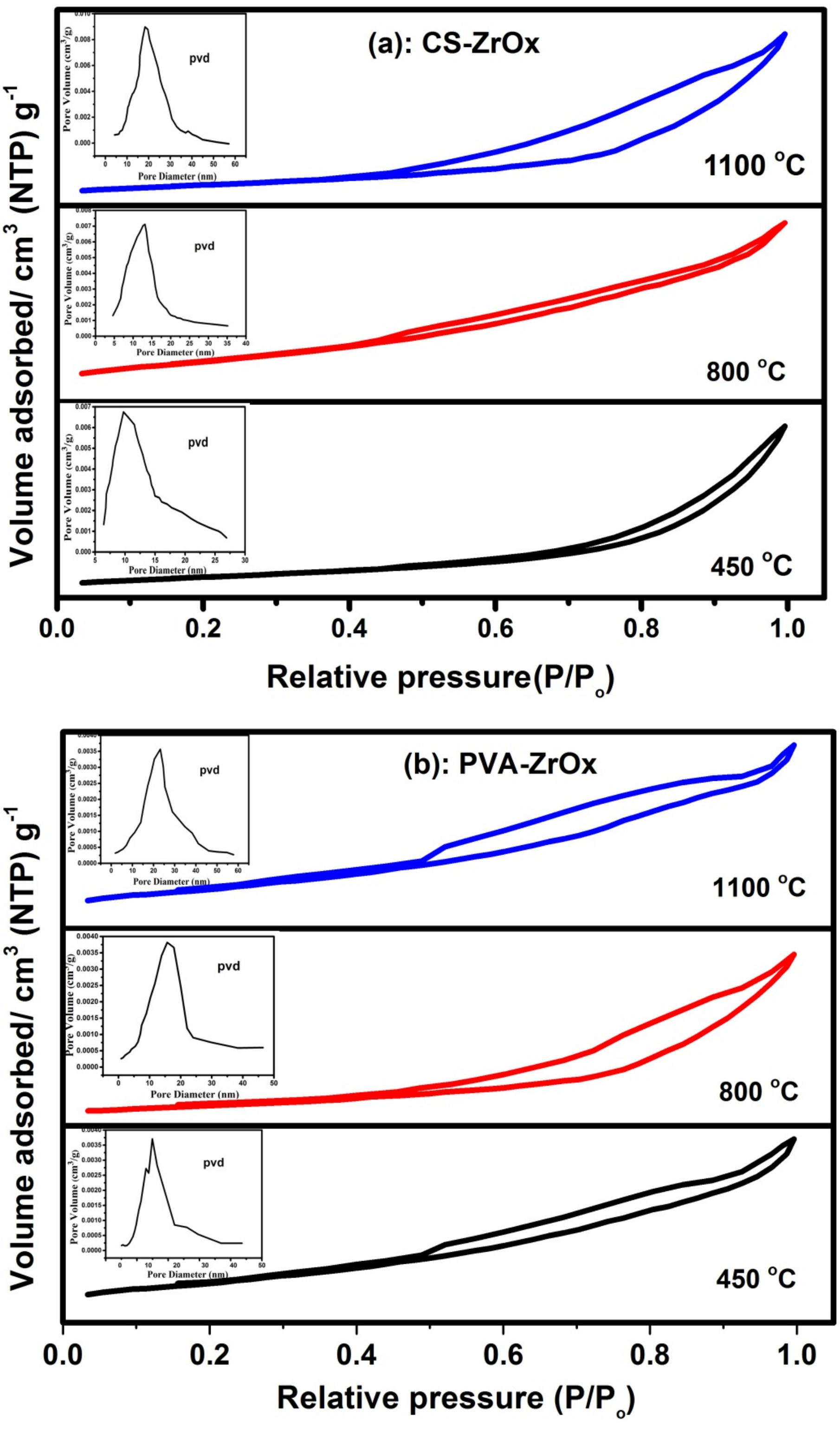

Figure S4. N_2 adsorption-desorption isotherm determined on ZrO_2 NPs recovered from CS-ZrOx (a) and PVA-ZrOx (b) hybrid films at the calcination temperatures indicated. Inset figures display corresponding pvd curves.

Table S1: Binding energies values (±0.02 eV) obtained by curve fitting of XPS Zr3d and O1s spectra measured on surfaces of ZrO_2 NPs recovered from CS-ZrOx and PVA-ZrO_x hybrid films at the calcination temperatures indicated.

**Hybrid film**	**Calcination temp./°C**	**$Zr3d_{5/2, 3/2}$ /eV**	**O1s /eV**
CS-ZrOx	450	182.0, 184.4	530.0, 531.5
	800	181.9, 184.3	529.9, 531.4
	1100	181.8, 184.4	529.8, 531.3
PVA-ZrOx	450	181.9, 184.3	529.9, 531.4
	800	181.8, 184.4	529.8, 531.3
	1100	181.7, 1.84.3	529.7, 531.3
